# Characterizing ovarian histopathology in transgender and gender-diverse patients on testosterone: Informing shared decision-making in gender-affirming care

**DOI:** 10.3389/frph.2026.1738050

**Published:** 2026-04-01

**Authors:** Kia Gianni Thigpen, Natalia Gontarczyk Uczkowski, Amy L. Godecker, Madison Seifer, Alexandra Sabgir, Laura Hanks

**Affiliations:** Department of Obstetrics and Gynecology, University of Wisconsin-Madison, Madison, WI, United States

**Keywords:** benign - ovarian histology without remarkable findings, gender-affirming surgery, gonadectomy, ovary, pathology - any change in ovarian histology other than benign, testosterone exposure - any subcutaneous, transdermal or subcutaneous administration of testosterone prior to time of surgery

## Abstract

**Background:**

Gender-affirming hysterectomies (GAH) are increasingly performed among transgender and gender-diverse (TGD) individuals, many of whom utilize testosterone. The World Professional Association for Transgender Health (WPATH) guidelines regarding oophorectomy at the time of hysterectomy defer to shared decision-making, as limited data exists on the effects of exogenous testosterone on an individual's ovaries. This study aims to describe the characteristics of the ovarian histopathology exemplified in this cohort and contribute to the growing gender-affirming care database that guides shared decision making between providers and patients that best align with a patient's mental and physical health goals.

**Methods:**

This observational study reviewed all hysterectomies performed in TGD individuals at a single academic institution in Wisconsin between January 1st, 2016 and December 31st, 2023 for primary or secondary indication of gender dysphoria. This study was submitted to the institutional review board (IRB) at the academic institution at which it was performed and was deemed exempt. Individuals were excluded if age <18, indication included malignancy, surgery was completed by an ObGyn subspecialist, or they had not used exogenous testosterone prior to surgery. Ovarian pathology reports and medical records for all subjects who underwent unilateral and bilateral oophorectomy at the time of their hysterectomy were reviewed, and patient demographics, indication for procedure, and preoperative testosterone use were abstracted.

**Results:**

During the study period, 144 hysterectomies were performed in self-identified TGD individuals. Of these, 95 (65.9%) underwent oophorectomy, either unilateral or bilateral, of which 89 (93.6%) of these hysterectomies plus oophorectomies had a primary or secondary indication of gender dysphoria/incongruence. One individual did not report prior use of exogenous testosterone and was excluded, leaving a study population size of 88 patients. Gender dysphoria/incongruence (84.1%) was the most common indication. Benign or unremarkable ovaries was the most common diagnosis in the 64 (72.8%) ovarian pathology reports. Simple/follicular cysts were seen in 20 (22.7%) of TGD individuals' ovarian specimens. No malignant ovarian pathology was identified in this cohort based on institutional procedural configuration, however, uncommon pathologies were also not identified presumably due to the sample size and age of the population studied.

**Conclusions:**

Ovarian pathology observed in this cohort of TGD individuals undergoing gender-affirming hysterectomy is consistent with previously reported findings, with benign ovarian changes being most common among TGD patients. However, rare outcomes and pathologies with long latency periods require substantially larger cohorts and longer follow-up to detect. As such, the absence of uncommon or malignant findings in this study should not be interpreted as evidence of low population risk. Rather, these results contribute incremental data to the limited body of evidence describing ovarian pathology in TGD individuals using testosterone.

## Introduction

Gender-affirming hysterectomies with oophorectomy are increasingly performed among transgender and gender-diverse (TGD) individuals (1). For individuals who desire this procedure, the World Professional Association for Transgender Health (WPATH) states it is considered medically necessary surgery in the process of gender affirmation (2). The decision of whether to pursue oophorectomy at the time of gender-affirming hysterectomy is nuanced given the multifunctional nature of ovaries and the implications of their removal (3).

Ovaries are important endocrine organs that produce hormones which are important for many physiologic functions. Hormone production from one ovary is sufficient to maintain their endocrinologic role, but removal of both ovaries in a premenopausal person necessitates hormone therapy to protect against the potential negative effects of early removal (4).

For cisgender women of premenopausal age, elective oophorectomy is not recommended at the time of hysterectomy for benign indications given that the long-term health effects of removal outweigh the potential benefits in someone who does not have an elevated risk of malignancy. However, for TGD individuals, the guidelines are less clear. WPATH advises shared decision-making between the physician and patient when deciding whether to pursue oophorectomy at the time of gender-affirming hysterectomy. This decision is multifaceted, as it can depend on other aspects of the individual's gender-affirmation journey and personal goals, including whether they utilize testosterone therapy, whether an estrogen depleted state would assist in aligning physical characteristics with gender identity and whether they would like to preserve fertility through ovarian retention. Additionally, an individual's decision to retain their ovaries may be due to concerns regarding access to exogenous hormone supplementation. This access could be discontinued due to financial, practical, legal, or medical reasons. Alternatively, one might opt to remove their ovaries if there is a family history of ovarian cancer.

The American College of Obstetricians and Gynecologists (ACOG) notes that not all TGD individuals experience gender dysphoria related to their ovaries and therefore hormone suppression via oophorectomy may not be desired (5). However, it is also understood that pelvic organs and secondary sex characteristics resulting from the hormones they produce may be a source of dysphoria and thoughtful discussion and planning can alleviate this sense of dysphoria in some individuals (6).

Many individuals who undergo gender-affirming hysterectomies are on long-term testosterone therapy. However, limited data exists on the effects of exogenous testosterone on ovaries, which makes it challenging for clinicians to appropriately counsel whether to undergo oophorectomy at the time of gender-affirming hysterectomy. In a large multicenter case series, Grimstad et al. found that for TGD individuals on long-term testosterone who underwent oophorectomy, there was no increased risk of ovarian malignancy. Benign pathology, in their cohort, suggested ovarian retention while on testosterone as an option for those desiring future fertility (7).

This study aims to contribute to the existing data available regarding ovarian histopathology in TGD persons. The objective is to characterize the ovarian findings in this cohort of TGD individuals.

## Methods

This is an observational study that reviewed all hysterectomies performed in TGD individuals at a single academic institution in Wisconsin between January 1st, 2016 and December 31st, 2023 for primary or secondary indication of gender dysphoria. Patients were identified using Current Procedural Terminology codes for hysterectomies based on International Classification of Diseases (ICD) codes IP-ICD-10-PCS and OP-CPT, both abdominal and vaginal routes. Using these codes the Slicer Dicer application within Epic was used to identify all hysterectomies performed for gender dysphoria or incongruence using ICD code ICD-10: F64. Two medical student researchers were responsible for chart abstraction with a supervising board-certified ObGyn responsible for affirming the abstracted charts.

Charts were reviewed to exclude those with retained bilateral ovaries. Those who had unilateral or bilateral oophorectomy at the time of hysterectomy were included. Individuals were excluded from the study if their age was less than 18, their indication for hysterectomy or oophorectomy included malignancy as this Wisconsin-based institution does not conduct hysterectomies for malignant indications, or their hysterectomy was completed by an ObGyn subspecialist (i.e., Gynecology Oncology, Urogynecology, Minimally Invasive Gynecologic Surgery and Maternal Fetal Medicine). Subspecialist cases were excluded to aid in reporting on standardized patients.

Pathologists from the institution reviewed specimens and had access to all charts at the time of their review. All charts that met criteria were included and their original pathology reports were reviewed by medical student researchers who documented the pathology statements in REDCap. The statements were systematically reviewed by a board-certified obstetrician and gynecologist (author LH) to codify the presence or absence of pathology into the following eight categories: simple/follicular cysts, corpus luteal cyst, teratoma or dermoid cyst, endometriomas or endometriosis cysts, fibroma, serous cystadenofibroma or serous cystadenoma, or benign/unremarkable, and “does not comment” which indicated the pathologist did not comment on the ovaries in their report. Biostatisticians reviewed these categories and documented the findings in table format. Neither the obstetrician and gynecologist nor the biostatisticians were blinded to testosterone exposure during their abstraction and table formation.

Details of testosterone use, including duration of use, route of administration, serum testosterone level prior to surgery, number of weeks prior to surgery that the pre-operative testosterone level, and whether testosterone therapy was discontinued before surgery were abstracted through manual chart review of patient reports. No data was obtained on gaps or pauses in testosterone use, formulation changes in the testosterone administered or general adherence. Thus, the definition of duration is an assumption of continued use from start date based on first ordered testosterone and patient report of administration through time of surgery. Additional data abstracted included demographics (age, gender and race) and surgical details such as route of hysterectomy and indication.

Statistical analysis was performed using SAS version 9.4. Descriptive statistics were used to characterize demographic variables, surgical details, testosterone usage and levels. A Monte Carlo estimation was used to test the association between the duration of exogenous testosterone and ovarian pathology. This study was submitted to the institutional review board (IRB) at the academic institution at which it was performed and was deemed exempt as a retrospective chart review.

## Results

**Figure 1:**
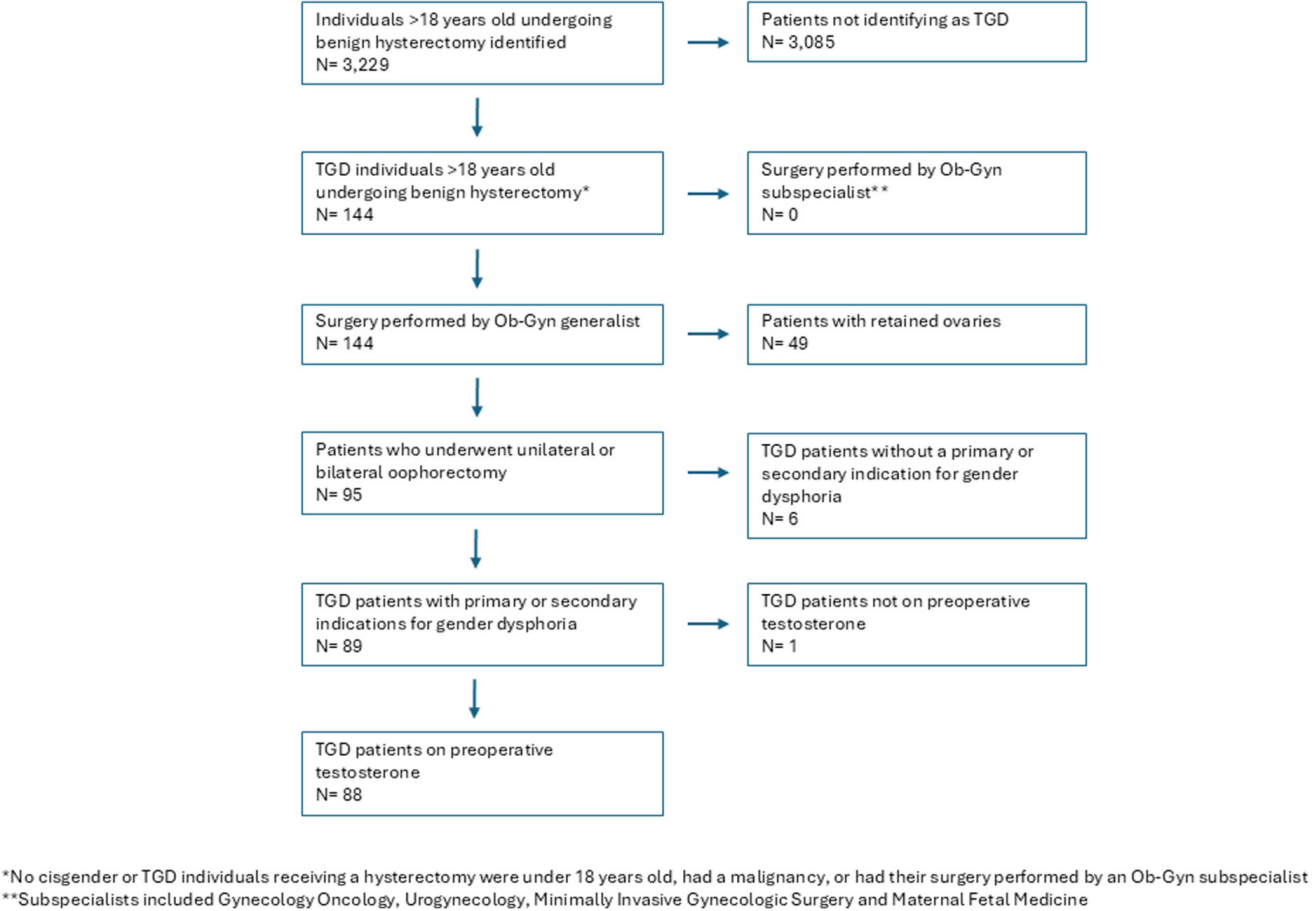
Flow diagram mapping included cases.

A total of 144 hysterectomies were performed for people who identified as TGD between January 1st, 2016 and December 31st, 2023 at a single-institution. Eighty-nine individuals underwent hysterectomy and either unilateral or bilateral oophorectomy. Of these individuals, one patient was excluded because they did not use exogenous testosterone prior to surgery, for a final cohort of 88 patients (Figure 1). The median age at hysterectomy with either unilateral or bilateral oophorectomy was 29.5 years (Table 1). Of this cohort of TGD patients, 83 (94.3%) identified as transgender males, and 5 (5.7%) identified as non-binary. The majority of patients identified as White (86.2%) followed by African American (6.9%) and Asian (4.6%). No patients identified as Native American or Pacific Islander. Seventy-four (84.1%) individuals underwent hysterectomy for gender dysphoria/incongruence as the primary indication. In addition, 8 (9.1%) and 5 (5.7%) individuals underwent hysterectomy for pelvic pain and abnormal uterine bleeding as the primary indication, respectively. An additional 14 patients (15.9%) had a secondary indication of gender dysphoria/incongruence for their hysterectomy.

**Table 1:** Demographic characteristics of study cohort.

Characteristic	Transgender and gender diverse (TGD) cohort (*n* = 88)
Age (y), mean (sd)	29.5 (7.8)
BMI (kg/m^2), mean (sd)	29.7 (7.2)
Gender, n(%) Cisgender female Trans-male Non-binary Other	0 (0.0) 83 (94.3) 5 (5.7) 0 (0.0)
Race, n(%) White African American Asian Native American Pacific Islander Other/not documented	75 (86.2) 6 (6.9) 4 (4.6) 0 (0.0) 0 (0.0) 2 (2.3)
Indication, n(%) Gender dysphoria/incongruence Abnormal uterine bleeding Fibroids Pelvic pain Cervical dysplasia Other	74 (84.1) 5 (5.7) 0 (0.0) 8 (9.1) 0 (0.0) 1 (1.1)
Secondary Indication, n(%) Gender dysphoria/incongruence Abnormal uterine bleeding Fibroids Pelvic pain Cervical dysplasia Other None	14 (15.9) 6 (6.8) 0 (0.0) 5 (5.7) 0 (0.0) 3 (3.4) 60 (68.2)
Ovarian pathologies present, n(%) Simple/Follicular cysts Corpus luteal cyst Teratoma or dermoid cyst Endometriomas or endometriotic cysts Fibroma Serous cystadenofibroma or serous cystadenoma Benign/unremarkable “Does not comment”	20 (22.7) 0 (0.0) 0 (0.0) 0 (0.0) 1 (1.1) 2 (2.3) 64 (72.8) 1 (1.1)

Benign or unremarkable were noted in 64 (72.8%) of ovarian pathology reports. Simple or follicular cysts were seen in 20 (22.7%). There were no corpus luteal, teratoma or dermoid cysts, endometriomas or endometriotic cysts in this study cohort.

The median duration of testosterone use in the study cohort was 41.5 months based on patient reports found during chart abstraction with interquartile range of 21.0, 67.0 (Table 2). Serum testosterone levels were obtained in 74 (84.1%) of individuals in this cohort with a mean serum testosterone level of 609.4 (sd 261.1). The majority of individuals in this cohort used intramuscular injections (69.3%) for route of testosterone administration.

**Table 2:** Preoperative testosterone use in the study cohort.

Characteristics	Transgender and gender diverse (TGD) cohort (*n* = 88)
Duration of testosterone use, n(%) 0–6 months 7–12 months 13–24 months 25–48 months >=49 months	2 (2.3) 4 (4.6) 18 (20.9) 31 (36.1) 31 (36.1)
Duration of testosterone use (months), median (IQR)	41.5 (21.0, 67.0)
Serum testosterone level obtained, n(%)	74 (84.1)
Weeks before surgery that pre-op testosterone was collected, median (IQR)	25.0 (10.0, 48.0)
Serum testosterone level (ng/dL), mean (sd)*	609.4 (261.1)
Route of testosterone use, n(%) Intramuscular Transdermal Subcutaneous	61 (69.3) 10 (11.4) 17 (19.3)
Testosterone discontinued before surgery, n(%)	1.0 (1.1)

* For individuals using intramuscular and subcutaneous injections, our institution recommends individuals have their labs drawn 3–4 days after their most recent injection.

Table 3 cross tabulates duration of testosterone use and ovarian pathology. There was no statistically significant association between duration of testosterone use and ovarian pathology (*p* = 0.536). Simple and follicular cysts were the most common finding across all duration groups. Other ovarian pathologies were rare.

**Table 3:** Ovarian pathology by duration of testosterone use.

	Duration of testosterone use:					
Ovarian pathologies n(%) [95% CI]	0–6 months (*n* = 2)	7–12 months (*n* = 4)	13–24 months (*n* = 18)	25–48 months (*n* = 31)	>=49 months (*n* = 31)	Monte Carlo estimation *p*-value
Simple/ Follicular cysts	1 (100.0) [100.0, 100.0]	1 (100.0) [100.0, 100.0]	2 (66.7) [9.0, 100.0]	10 (90.9) [72.5, 100.0]	6 (85.7) [57.7, 100.0]	0.536
Corpus luteal cyst	0 (0.0) [-]	0 (0.0) [-]	0 (0.0) [-]	0 (0.0) [-]	0 (0.0) [-]	
Teratoma or dermoid cyst	0 (0.0) [-]	0 (0.0) [-]	0 (0.0) [-]	0 (0.0) [-]	0 (0.0) [-]	
Endometriomas or endometriotic cysts	0 (0.0) [-]	0 (0.0) [-]	0 (0.0) [-]	0 (0.0) [-]	0 (0.0) [-]	
Fibroma	0 (0.0) [-]	0 (0.0) [-]	0 (0.0) [-]	1 (9.1) [0.0, 27.5]	0 (0.0) [-]	
Serous cystadenofibroma or serous cystadenoma	0 (0.0) [-]	0 (0.0) [-]	1 (33.3) [0.0, 91.0]	0 (0.0) [-]	1 (14.3) [0.0, 42.3]	

In a sub-analysis of the five non-binary participants, all individuals had documented serum testosterone levels, with a median duration of testosterone therapy of 41 months (IQR 32–44) and median levels of 672 ng/dL (IQR 629–857) (Table 4). Their testosterone regimens included intramuscular (40%) and subcutaneous (60%) routes, and histopathologic findings were predominantly benign, with four individuals (80%) demonstrating unremarkable ovarian tissue and one individual (20%) exhibiting a simple or follicular cyst, mirroring the distribution of benign findings observed in the larger transgender cohort.

**Table 4:** Preoperative testosterone use for non-binary and transgender patients.

Characteristics	Non-binary patients (*n* = 5)	Transgender patients (*n* = 83)	*p*-value
Age, median (IQR)	34.0 (33.0, 36.0)	28.0 (23.0, 34.0)	0.127*
Duration of testosterone use, n(%)[95% CI] 0–6 months 7–12 months 13–24 months 25–48 months >=49 months	0 (0.0) [-] 0 (0.0) [-] 1 (20.0) [0.0, 55.8] 3 (60.0) [16.2, 100.0] 1 (20.0) [0.0, 55.8]	2 (2.5) [0.0, 5.9] 4 (4.9) [0.1, 9.8] 17 (21.0) [11.9, 30.0] 28 (34.6) [24.0, 45.1] 30 (37.0) [26.3, 47.8]	0.888**
Duration of testosterone use (months), median (IQR)	41.0 (32.0, 44.0)	42.0 (21.0, 67.0)	0.883***
Serum testosterone level obtained, n(%)[95% CI]	5 (100.0) [100.0, 100.0]	69 (83.1) [74.9, 91.3]	>0.999**
Weeks before surgery that pre-op testosterone was collected, median (IQR)	17.0 (16.0, 20.0)	25.0 (10.0, 48.0)	0.883***
Serum testosterone level, median (IQR)	672.0 (629.0, 857.0)	587.0 (423.0, 720.0)	0.883***
Route of testosterone use, n(%)[95% CI] Intramuscular Transdermal Subcutaneous	2 (40.0) [0.0, 83.8] 0 (0.0) [-] 3 (60.0) [16.2, 100.0]	59 (71.1) [61.1, 81.0] 10 (12.0) [4.9, 19.2] 14 (16.9) [8.7, 25.1]	0.067**
Testosterone discontinued before surgery, n(%)[95% CI]	0 (0.0) [-]	1 (1.2) [0.0, 3.6]	>0.999**
Ovarian pathologies present, n(%)[95% CI] Simple/Follicular cysts Fibroma Serous cystadenofibroma or serous cystadenomas Benign/unremarkable Does not comment)	1 (20.0) [0.0, 55.8] 0 (0.0) [-] 0 (0.0) [-] 4 (80.0) [44.2, 100.0] 0 (0.0) [-]	19 (22.9) [13.7, 32.1] 1 (1.2) [0.0, 3.6] 2 (2.4) [0.0, 5.8] 60 (72.3) [62.5, 82.1] 1 (1.2) [0.0, 3.6]	>0.999**

* Mann–Whitney U test.

** Fisher's exact.

*** Mann–Whitney.

## Discussion

This study is an incremental addition to the growing body of literature evaluating the impact of exogenous testosterone on ovarian pathology in TGD individuals undergoing gender-affirming hysterectomy. Our findings demonstrate that most of the ovarian pathology in this TGD cohort was benign or unremarkable, regardless of duration of testosterone use.

The most common findings in our cohort were benign ovarian histology or simple or follicular cysts, mirroring the studies of Cao et al. (8) and Kumar et al. (9), who report a predominance of non-pathologic or benign findings in TGD patients (8, 9). These findings may suggest long-term testosterone therapy does not induce pathologic changes in ovarian tissue.

Duration of testosterone use did not impact the type or frequency of benign ovarian pathology. Grimstad et al. (7) previously noted that ovarian changes such as stromal hyperplasia or luteinized follicles can be seen in patients on testosterone (6). These are considered physiologic or hormonally induced changes, not malignant precursors, which support the use of testosterone as hormonal suppression in TGD individuals who desire to preserve their ovaries.

Broader findings from the literature reinforce that the ovarian morphology observed in our cohort parallels what has been consistently described in individuals on testosterone therapy. Across multiple histopathologic studies—including case reports, case series, descriptive studies, and comparative analyses—ovaries from testosterone-exposed individuals most commonly show multicystic architecture**,** thickening of the tunica albuginea**,** stromal hyperplasia, and foci of luteinized stromal cells (10–17). These features have been documented in both earlier studies of long-term androgen exposure and in more recent cohorts, and reflect hormonally mediated changes rather than true pathologic alterations.

Physiologic ovarian activity may persist despite exogenous testosterone, as multiple studies have identified corpora lutei, including recent or hemorrhagic corpora lutea, suggesting ongoing or intermittent ovulation (10). These findings support the concept that testosterone does not universally suppress ovulation and may help explain preserved fertility potential in some individuals on long-term testosterone therapy.

Although the overwhelming majority of reported findings are benign, rare cases of ovarian neoplasia have been described. These include isolated reports of serous papillary carcinomas**,** borderline serous tumors, a well-differentiated endometrioid adenocarcinoma, and a small incidental Brenner tumor (18–20). Notably, these cases remain extremely uncommon and do not indicate a causal relationship between testosterone therapy and ovarian malignancy—particularly when considered against the large body of evidence showing no increased malignancy rates in testosterone-exposed ovaries. In this context, the isolated reports underscore the importance of routine pathologic evaluation but do not contradict the broader conclusion that exogenous testosterone does not confer increased neoplastic risk.

The sub-analysis of participants who identified as non-binary revealed patterns of testosterone use and largely benign ovarian histopathology that were consistent with those of the broader transgender cohort. Although limited by small sample size, these findings contribute to the growing body of evidence indicating that ovarian pathology does not differ meaningfully across gender-diverse individuals receiving masculinizing hormone therapy, reinforcing the overall safety profile of long-term testosterone use in this context.

Overall, integration of our findings with the existing literature demonstrates a highly consistent pattern: ovarian changes associated with testosterone hormone therapy are overwhelmingly benign reinforcing the safety of testosterone therapy for TGD individuals who elect to retain their ovaries.

Limitations of the study include its retrospective and single-institution design which hinders its generalizability and external validity. There was one obstetrician and gynecologist who reviewed all pathology reports which is a limitation as inter-rater agreement was not obtained though the biostatisticians did offer agreement when constructing the final tables. Additionally, the study is underpowered to detect an association with testosterone use and more uncommon pathologies like endometriomas. The institution where this study took place also does not perform hysterectomies for malignant indications. This removes any cases of TGD individuals with malignancy as an indication for hysterectomy from our study and could be a future direction for comparison. A control group was not included in this study as the aim was to be descriptive and many cisgender individuals undergoing hysterectomy with and without oophorectomy are generally older and have more comorbidities than this cohort. This may be viewed as a limitation as it narrows the generalizability of the study. Additionally, cases with subspecialty involvement during this particular observed time period were excluded. Subspecialists typically perform more complex surgeries and thus not having these cases reflected in the study may limit observing more complex pathology.

The strengths of this study include the number of patients examined and the detailed pathology reports reviewed. The findings are consistent with prior research available and offer a nuanced response to an area of gender affirming care that lacks robust data. The strength lies in the implications of majorly benign findings to clinical counseling and surgical decision-making. Given this study's sample size, the benign nature of the removed ovaries suggests this cohort's ovaries were marginally impacted by testosterone use. Further data with larger cohort sizes could imply a benign ovarian profile for those who desire future fertility (via egg retrieval and surrogacy), wish to avoid early oophorectomy and its possible sequelae, or do not experience dysphoria related to their ovaries. For patients who seek elective oophorectomy as part of their gender-affirmation process, these findings reinforce an individualized, shared decision-making framework to best align with a patients' mental and physical health goals.

## Conclusion

Ovarian pathology in TGD individuals undergoing gender-affirming hysterectomy were overall benign or unremarkable. These findings provide reassurance to patients and clinicians considering ovarian preservation or elective oophorectomy at the time of gender-affirming hysterectomy.

The predominance of benign or unremarkable pathology in this cohort of TGD individuals on testosterone supports either a clinically conservative approach of ovarian preservation or removal. The consistency of our results with existing literature strengthens the recommendation to individualize oophorectomy decisions, rather than adopt a default approach of routine removal or preservation during hysterectomy in this population. Multi-center, long-term, prospective studies are needed to further validate these conclusions. Additional studies analyzing how testosterone duration impacts ovarian histopathology could further the understanding of ovarian preservation. Another future direction to strengthen counseling would be to evaluate the long-term effects to bone, heart, and brain health in TGD patients who opt to retain or remove their ovaries.

Finally, although our findings and prior studies consistently demonstrate a predominance of benign or unremarkable ovarian pathology in TGD individuals on testosterone therapy, we believe routine pathologic evaluation of all excised ovarian tissue remains important. While some have suggested that such evaluation could be omitted in resource-limited settings or when patients bear the cost, our study is not sufficiently powered to conclude that omission is safe. Rare pathologic findings, though uncommon, may have clinical significance, and histologic review ensures comprehensive patient care and quality assurance.

## Data Availability

The original contributions presented in the study are included in the article, further inquiries can be directed to the corresponding author.
